# Efficient and precise base editing in rabbits using human APOBEC3A-nCas9 fusions

**DOI:** 10.1038/s41421-019-0099-5

**Published:** 2019-06-11

**Authors:** Zhiquan Liu, Siyu Chen, Huanhuan Shan, Quanjun Zhang, Mao Chen, Liangxue Lai, Zhanjun Li

**Affiliations:** 1 Key Laboratory of Zoonosis Research, Ministry of Education, College of Animal Science, Jilin University, Changchun, 130062 China; 2 CAS Key Laboratory of Regenerative Biology, Guangdong Provincial Key Laboratory of Stem Cell and Regenerative Medicine, South China Institute for Stem Cell Biology and Regenerative Medicine, Guangzhou Institutes of Biomedicine and Health, Chinese Academy of Sciences, Guangzhou, 510530 China; 3 Guangzhou Regenerative Medicine and Health Guang Dong Laboratory (GRMH-GDL), Guangzhou, 510005 China; 4 Institute for Stem Cell and Regeneration, Chinese Academy of Sciences, Beijing, 100101 China

**Keywords:** Genomic analysis, Double-strand DNA breaks

Dear Editor:

The largest class of known human genetic diseases are caused by point mutations or single-nucleotide polymorphisms (SNPs)^1^. Base editors that can achieve targeted C-to-T conversions without generating DNA double-strand breaks, or requiring a donor template, represent significant advances in both disease modeling and gene therapy^2^. However, the currently used base editor 3 (BE3), derived from the rAPOBEC1 (rA1)-nCas9 (Cas9 nickase, D10A) fusion, is limited by low editing efficiency in GC contexts, and by high bystander activity when more than one C is present in the editing window^2,3^. Recently, human APOBEC3A (hA3A) was substituted for rA1 to produce new base editors (Fig. 1a). The editing window for hA3A-BEs was expanded to ~12 nt in human cells, and ~17 nt in plants and editing efficiency increased in GC contexts^4,5^. An engineered hA3A variant, hA3A-Y130F, had a limited editing window, similar to that of BE3 (~6 nt in human cells)^4,6^. Another variant, hA3A-N57G, showed a striking preference for the TCR (A/G) motif and a reduction in bystander mutations^7^. In this study, we demonstrate the effectiveness of the hA3A-nCas9 fusion and its associated variants (Y130F and N57G) to induce efficient and precise base editing in rabbits.

**Fig. 1:**
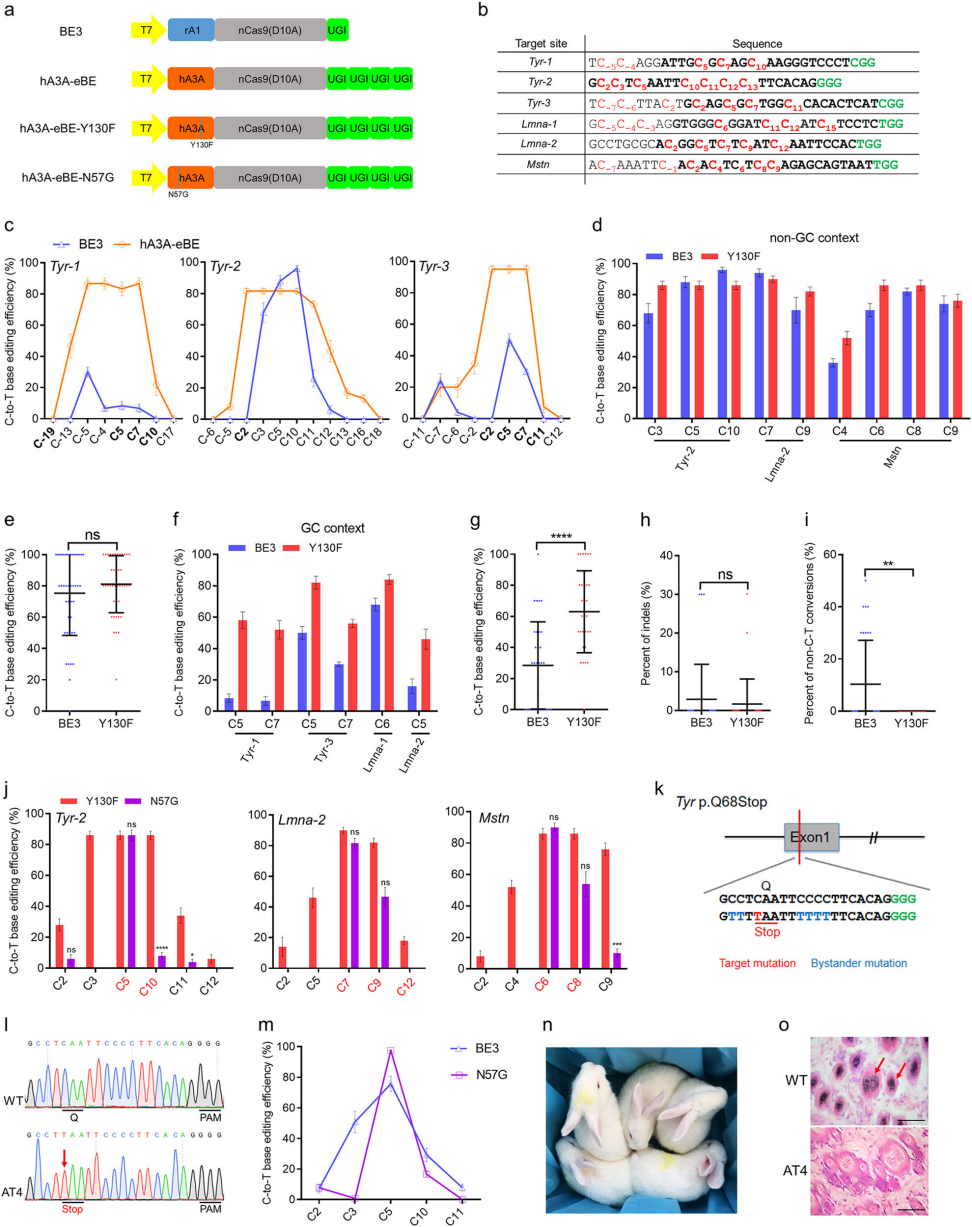
hA3A-nCas9 fusions induce efficient and precise C-to-T base editing in vivo. **a** Schematic representation of BE3 and hA3A-nCas9 fusion architecture. **b** Target-site sequences used in this study. Target sequence (black), sgRNA (bold), PAM region (green), target Cs (red, counting the PAM as positions 21–23). WT, wild-type. **c** Frequencies of single C-to-T conversions using BE3 and hA3A-eBE at three sites in rabbit embryos. GC contexts are indicated in bold on the *x* axis. **d, f** Comparison of C-to-T editing efficiency induced by BE3 and hA3A-eBE-Y130F in the target sites in non-GC context (**d**) or GC context (**f**). **e, g** Statistical analysis of the C-to-T editing frequency induced by BE3 and hA3A-eBE-Y130F in (**d**), (**f**), respectively. Forty-five target Cs for BE3 and 45 target Cs for hA3A-eBE-Y130F (**e**). Thirty-two target Cs for BE3 and 30 target Cs for hA3A-eBE-Y130F (**g**). **h, i** Statistical analysis of indels (**h**) or non-C-to-T conversions (**i**) induced by BE3 and hA3A-eBE-Y130F in rabbit embryos. Thirty-one embryos for BE3 and 30 embryos for hA3A-eBE-Y130F. **j** Frequencies of targeted single C-to-T conversions introduced by hA3A-eBE-Y130F and hA3A-eBE-N57G at three target sites in rabbit embryos. The TC contexts are indicated in red on the *x* axis. **k** The target sequence at the *Tyr* p. Q68Stop locus. The PAM and sgRNA target sequences are shown in green and black, respectively. Target mutation (red), bystander mutation (blue). **l** Representative Sanger sequencing chromatograms from a WT, and mutant rabbit (AT4). The red arrow indicates the substituted nucleotide. The relevant codon identities at the target sites are presented beneath the DNA sequence. **m** Comparison of the editing efficiency of single C-to-T conversions between BE3 and hA3A-eBE-N57G, at *Tyr* Q68Stop in F0 rabbit. The efficiency data of BE3 comes from our previous report^11^. **n** All five F0 rabbits exhibited a systemic albino phenotype. **o** H&E staining of skin from WT and *Tyr* mutant (AT4) rabbits. The red arrow highlights the melanin in the basal layer of the epidermis. Scale bars: 50 μm

We first tested hA3A-eBE in rabbit embryos at three target sites where GC (*Tyr-1* and *Tyr-3*) or non-GC (*Tyr-2*) contexts are contained in the editing window (Fig. 1b). As observed in human cells and plants^4,5^, hA3A-eBE showed a significantly expanded base editing window (~20 nt), compared with that of BE3 (~8 nt), and a higher editing efficiency in the GC context at *Tyr-1* and *Tyr-3* in rabbit blastocysts (Fig. 1c; Supplementary Figs. S1–3). Moreover, the formation of by-products including indels and non-C-to-T conversions by hA3A-eBE was slightly reduced compared to that by BE3 (Supplementary Fig. S4). These results indicated that hA3A-eBE can induce efficient C-to-T base editing with a large editing window and, independently of context, it could increase genome-targeting scope^8^.

Next, we examined the ability of hA3A-eBE-Y130F (narrowed editing window^4^) to perform base editing in rabbit embryos. A total of six single guide RNAs (sgRNA) containing GC and non-GC contexts were selected to evaluate the editing efficiency of hA3A-eBE-Y130F (Fig. 1b). The results showed that the primary deamination window for hA3A-eBE-Y130F spanned 7 nt (from protospacer positions 3 to 9), and is similar to that of BE3 in rabbit embryos (Supplementary Figs. S1–3 and S5–8). The genomic DNA was edited at all target sites, with the efficiency of hA3A-eBE-Y130F being slightly higher than that of BE3 (81.11 ± 2.72% versus 75.33 ± 4.02%) in non-GC contexts (Fig. 1d, e), consistent with reported findings in human cells and mice^4,6^. Within the editing window, in GC contexts, hA3A-eBE-Y130F induced significantly higher editing efficiencies than BE3 (63.00 ± 4.82% versus 28.44 ± 4.96%, *p* < 0.0001) (Fig. 1f, g). At all target sites, hA3A-eBE-Y130F-induced non-C-to-T conversions were significantly reduced (0.00 ± 0.00% versus 10.32 ± 3.02%, *p* < 0.01), but not indels, when compared with BE3 (Fig. 1h, i). The average frequency of by-products is relatively low using BE3, but in some samples, it reached 40–50%. This is consistent with the relatively high by-product frequency in individual mice and rabbit embryos using BE3 in previous studies^6,9–11^. Overall, these results demonstrated that hA3A-eBE-Y130F could induce efficient C-to-T base editing in rabbit embryos with high product purity, independently of sequence context.

The editing activities of an engineered base editor, hA3A-N57G, was high on cytidines in TCR (A/G) motifs, but greatly reduced when cytidines are in other sequence contexts^7^. Thus, to further improve precision in the TC context, a N57G mutation was introduced into hA3A-eBE to create hA3A-eBE-N57G (Fig. 1a). Three sgRNAs containing both TC and non-TC contexts were then selected for testing (Fig. 1b, j). The editing window for hA3A-eBE-N57G spanned ~5–9 nt, which is similar to that observed in human cells^7^, but the editing efficiency was significantly reduced in adjacent non-TC contexts, at all tested sites (Fig. 1j).

It is known that the *Tyr* gene is the major causal gene of human oculocutaneous albinism (OCA)^12^. The high frequency of bystander mutations (86%) was determined in the founder (F0) rabbits using BE3 in our previous study^11^. Thus, to generate a rabbit model to precisely mimic OCA, the mRNA encoding hA3A-eBE-N57G and sgRNA target to *Tyr* p.Q68Stop were injected into zygotes, making desired mutations in the TC context adjacent to multiple Cs (Fig. 1k). Consequently, five pups were obtained after injection and transplantation (Supplementary Table S1). The genotypes of the pups were determined using Sanger sequencing and deep sequencing. The results showed that all of them (100%) carried homozygous p.Q68Stop mutation (Figs. 1l and S9). Strikingly, hA3A-eBE-N57G significantly reduced bystander mutations of *Tyr* p.Q68Stop mutation in a TC context compared with that of the BE3 system (28% versus 86%) in founder rabbits (Supplementary Table S1). Further statistical analysis demonstrated the efficiency and precision of the hA3A-eBE-N57G-mediated mutation of *Tyr* p.Q68Stop, which has significantly fewer bystander mutations than does the BE3 system (target C5 on TC context, 97.60 ± 0.40% versus 75.67 ± 12.25%; bystander C3 in CC context, 0.80 ± 0.49% versus 50.83 ± 17.33%) (Fig. 1m). Moreover, a low proportion of indels or non-C-to-T mutations were detected in the F0 rabbits (Supplementary Fig. S9), consistent with a previous study using BE3^11^. Consistent with their genotypes, all five F0 rabbits exhibited a completely albino phenotype (Fig. 1n). Furthermore, hematoxylin and eosin (H&E) staining revealed the absence of melanin in the skin of mutant rabbits, compared with WT rabbits (Fig. 1o). In addition, deep sequencing was carried out to evaluate the off-target effect of the representative AT4 mutant. No obvious off-target mutations were detected at potential off-target sites (Supplementary Fig. S10), demonstrating that the hA3A-eBE-N57G system can mediate efficient base editing on the TCR (A/G) motif with high specificity in vivo.

In summary, we demonstrated that hA3A-eBE can induce efficient C-to-T conversions with a large editing window, and that hA3A-eBE-Y130F, having a narrowed editing window, can function as a generic version of the base editor, particularly at pathogenic SNPs in GC contexts. Furthermore, hA3A-eBE-N57G can induce efficient base editing in the TC context and minimize bystander mutations in vivo. Thus, these three APOBEC3A-nCas9 base editors expand the scope and improve the precision of the currently used BE3 system.

## Supplementary information

Supplementary information

## Supplementary information

**Supplementary Information** accompanies the paper at (10.1038/s41421-019-0099-5).
