# Supplementary material for: Efficient and precise base editing in rabbits using human APOBEC3A-nCas9 fusions
Source: Cell Discov. 2019 Jun 11;5:31. doi: 10.1038/s41421-019-0099-5 (PMC6557807; doi:10.1038/s41421-019-0099-5)
Supplement: Supplementary file 1 — Supplementary information [file 41421_2019_99_MOESM1_ESM.pdf]

Materials and Methods

Plasmid construction

pCMV-BE3 was obtained from Addgene (#73021). The hA3A-eBE and hA3A-eBE-Y130F plasmids were kind gifts from Xingxu Huang, Jia Chen and Li Yang[4]. The N57G mutation in hA3A was generated to produce hA3A-eBE-N57G. Plasmid site-directed mutagenesis was performed using the Fast Site-Directed Mutagenesis Kit (TIANGEN, Beijing). The site-directed mutation primers are listed in Supplementary Table S2.

mRNA and gRNA preparation

All plasmids were linearized with NotI and transcribed in vitro using the HiScribe™ T7 ARCA mRNA kit (NEB). mRNA was purified using the RNeasy Mini Kit (Qiagen) according to the manufacturer's protocol. sgRNA oligos were annealed into pUC57-sgRNA expression vectors containing a T7 promoter. The sgRNAs were then amplified and transcribed in vitro using the MAXIscript T7 kit (Ambion) and purified using the miRNeasy Mini Kit (Qiagen) according to the manufacturer's protocol. The sgRNA oligo sequences used in this study are listed in Supplementary Table S3.

Microinjection of rabbit zygotes

The protocol used for the microinjection of pronuclear-stage embryos has been described in detail in our previously published study.[1] Briefly, a mixture of mRNA (200 ng/ul) and sgRNA (50 ng/ul) was co-injected into the cytoplasm of pronuclear-stage zygotes.

Single-embryo PCR amplification and rabbit genotyping

The injected embryos were collected at the blastocyst stage. Genomic DNA of each embryo was extracted with an embryo lysis buffer (1% NP40) at 56 °C for 60 minutes and 95 °C for 10 minutes in a Bio-Rad PCR Amplifier. The genomic region surrounding the target site was PCR amplified and cloned into the pGM-T vector (TIANGEN). Both the PCR products and the colonies were sequenced. Genomic DNA was extracted from ear clips of newborn rabbits. The genomic regions surrounding the target site were PCR amplified and then subjected to both Sanger

sequencing and deep sequencing. Targeted sites were amplified from genomic DNA using Phusion polymerase (Thermo Fisher Scientific). Paired-end sequencing of PCR amplicons was performed by Sangon Biotech (Shanghai) using an Illumina MiSeq. All the primers for detection are listed in Supplementary Table S2.

Off-target assay

The potential off-target sites (POTs) with up to 3-nucleotide mismatches in the rabbit genome for sgRNA were predicted to analyse site-specific edits according to Cas-OFFinder (http://www.rgenome.net/cas-offinder/).[2] Deep sequencing was carried out to analyse the PCR products of the POTs. All the primers for the off-target assay are listed in Supplementary Table S4.

Haematoxylin and eosin (H&E) staining

The dorsal skin from WT and mutant rabbits was fixed in 4% paraformaldehyde for 48 hours, embedded in paraffin wax and then sectioned for slides. Slides were stained with haematoxylin and eosin (H&E) and viewed under a Nikon ts100 microscope.

Statistical analysis

All data are expressed as the mean ± SEM, with at least three individual determinations in all experiments. The data were analysed with t-tests using GraphPad prism software 6.0. A probability of $p < 0.05$ was considered statistically significant. $^{*}p < 0.05$, $^{**}p < 0.01$, $^{***}p < 0.001$, $^{****}p < 0.0001$.

Data availability

Sequencing data from this work has been deposited at the Sequence Read Archive under accession code SRP192541. The authors state that all data necessary for confirming the conclusions presented in this article are represented fully within the article, or can be provided by the authors upon request.

Ethics statement

New Zealand white and Lianshan black rabbits were obtained from the Laboratory Animal Center of Jilin University (Changchun, China). All animal studies were conducted according to experimental practices and standards approved by the Animal Welfare and Research Ethics Committee at Jilin University.

References for Materials and Methods

1. Song, Y. et al. Efficient dual sgRNA-directed large gene deletion in rabbit with CRISPR/Cas9 system. *Cell. Mol. Life Sci.* **73**, 2959–2968 (2016).

2. Bae, S., Park, J. & Kim, J. S. Cas-OFFinder: a fast and versatile algorithm that searches for potential off-target sites of Cas9 RNA-guided endonucleases. *Bioinformatics* **30**, 1473–1475 (2014).

Supplementary Figures

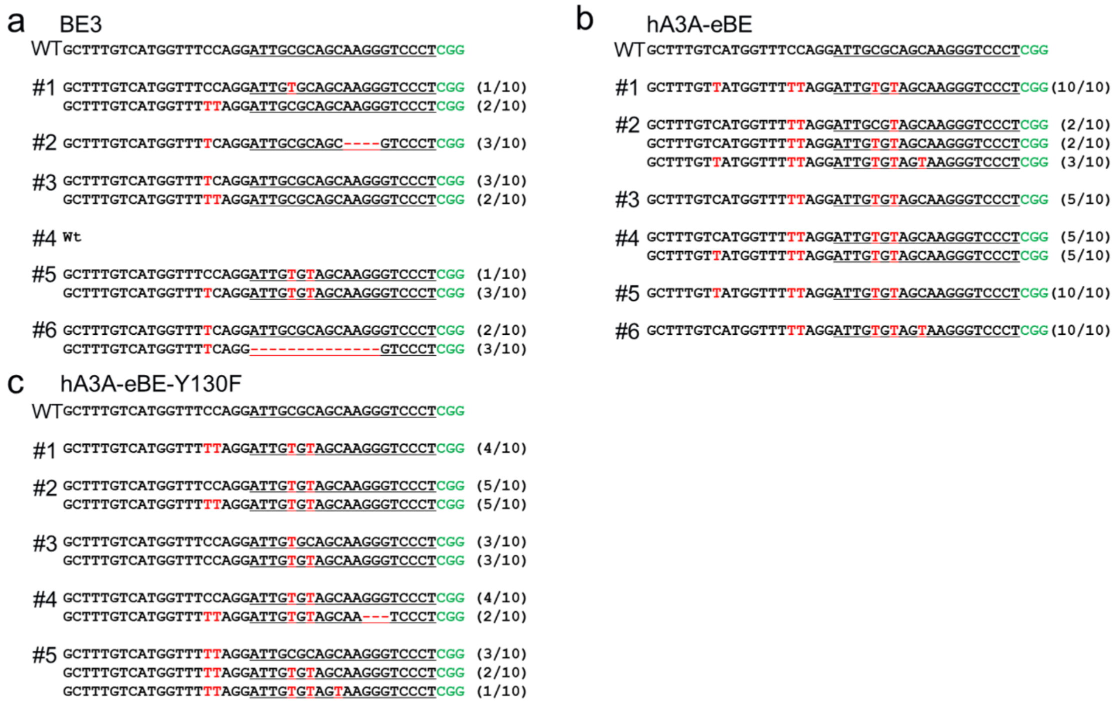

Figure S1. Comparison of the editing efficiencies of BE3 and A3A-Cas9 fusions at *Tyr-1* in rabbit blastocysts. The number of clones for each sequence pattern is indicated. The target sequence (underlined), PAM region (green) and substituted nucleotides (red) are shown. WT: wild-type. #1-#6: each blastocyst used for T-A cloning.

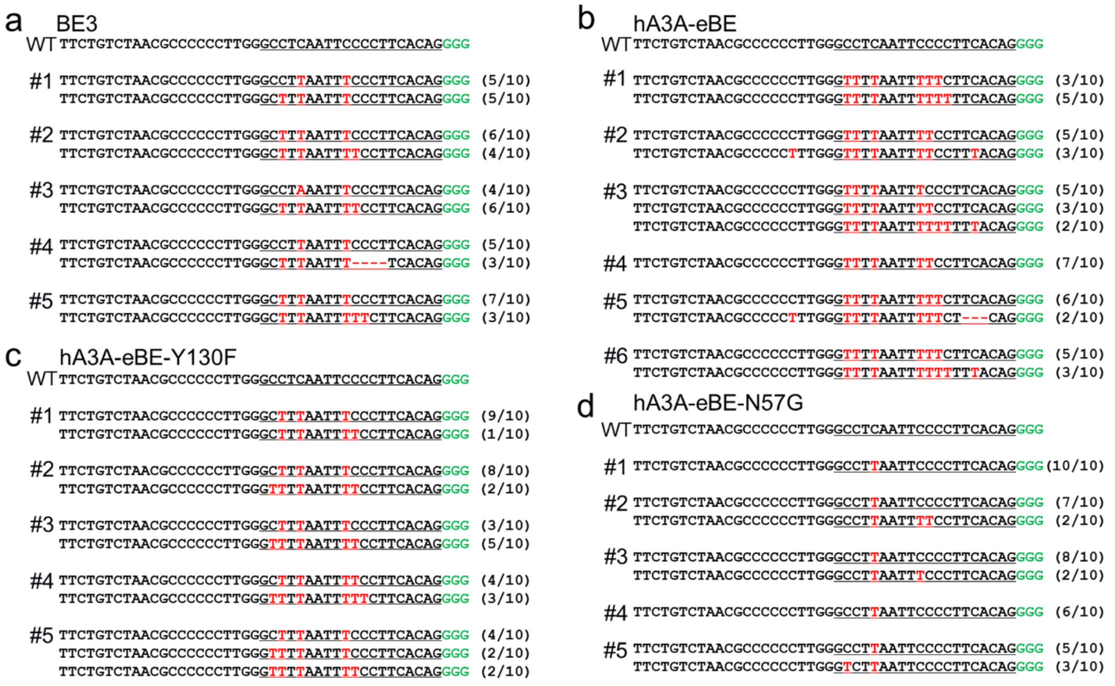

Figure S2. Comparison of the editing efficiencies of BE3 and A3A-Cas9 fusions at *Tyr-2* in rabbit blastocysts. The number of clones for each sequence pattern is indicated. The target sequence (underlined), PAM region (green) and substituted nucleotides (red) are shown. WT: wild-type. #1-#6: each blastocyst used for T-A cloning.

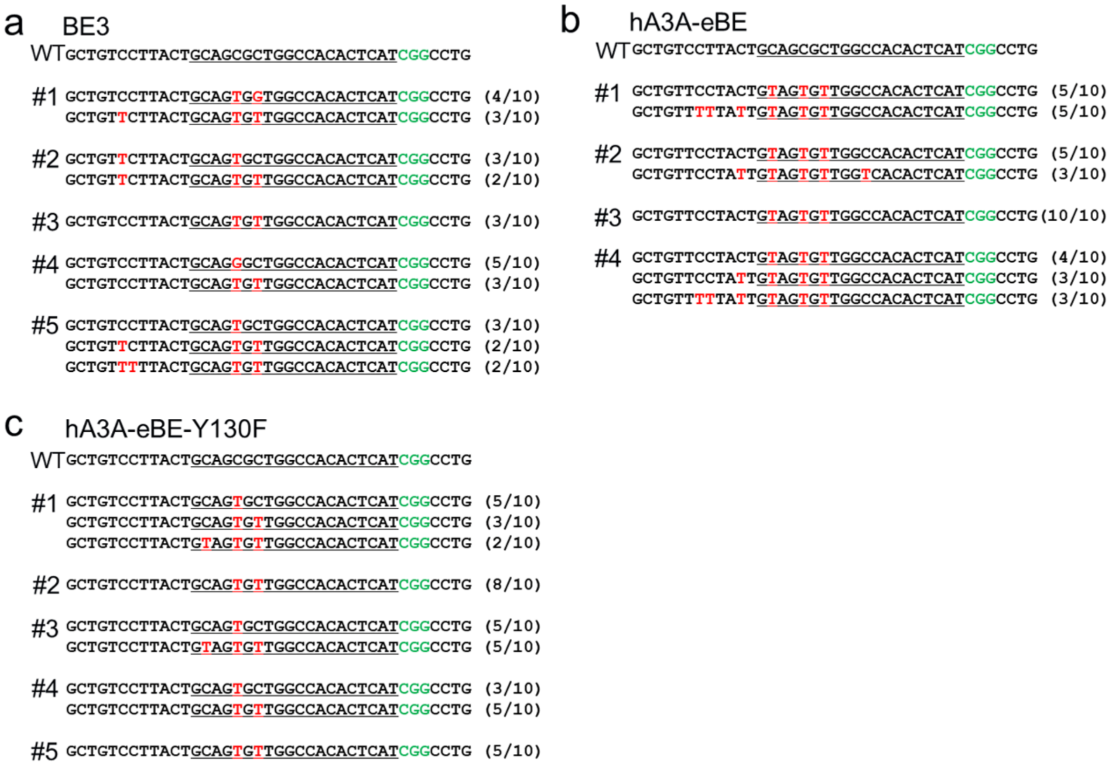

Figure S3. Comparison of the editing efficiencies of BE3 and A3A-Cas9 fusions at *Tyr-3* in rabbit blastocysts. The number of clones for each sequence pattern is indicated. The target sequence (underlined), PAM region (green) and substituted nucleotides (red) are shown. WT: wild-type. #1-#6: each blastocyst used for T-A cloning.

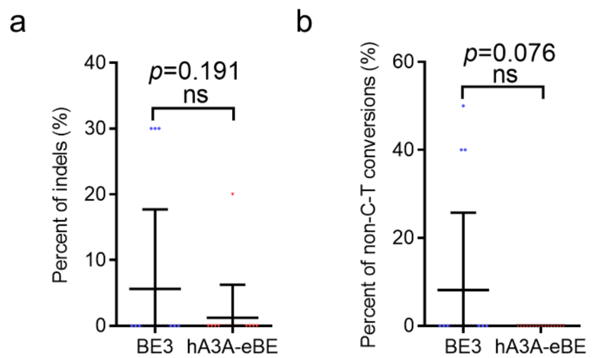

Figure S4. Comparison of the frequencies of by-product formation (including indels (a) and non-C-T conversions (b)) using BE3 and the hA3A-eBE system.

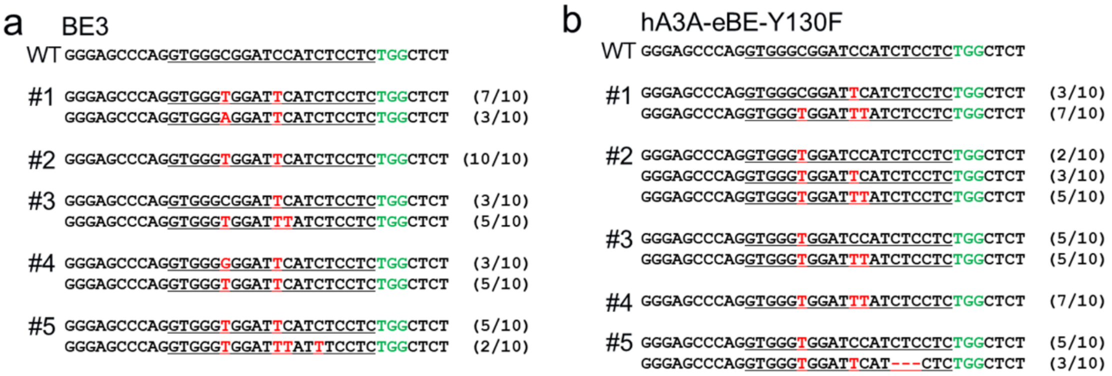

Figure S5. Comparison of the editing efficiencies of BE3 and A3A-Cas9 fusions at *Lmna-1* in rabbit blastocysts. The number of clones for each sequence pattern is indicated. The target sequence (underlined), PAM region (green) and substituted nucleotides (red) are shown. WT: wild-type. #1-#5: each blastocyst used for T-A cloning.

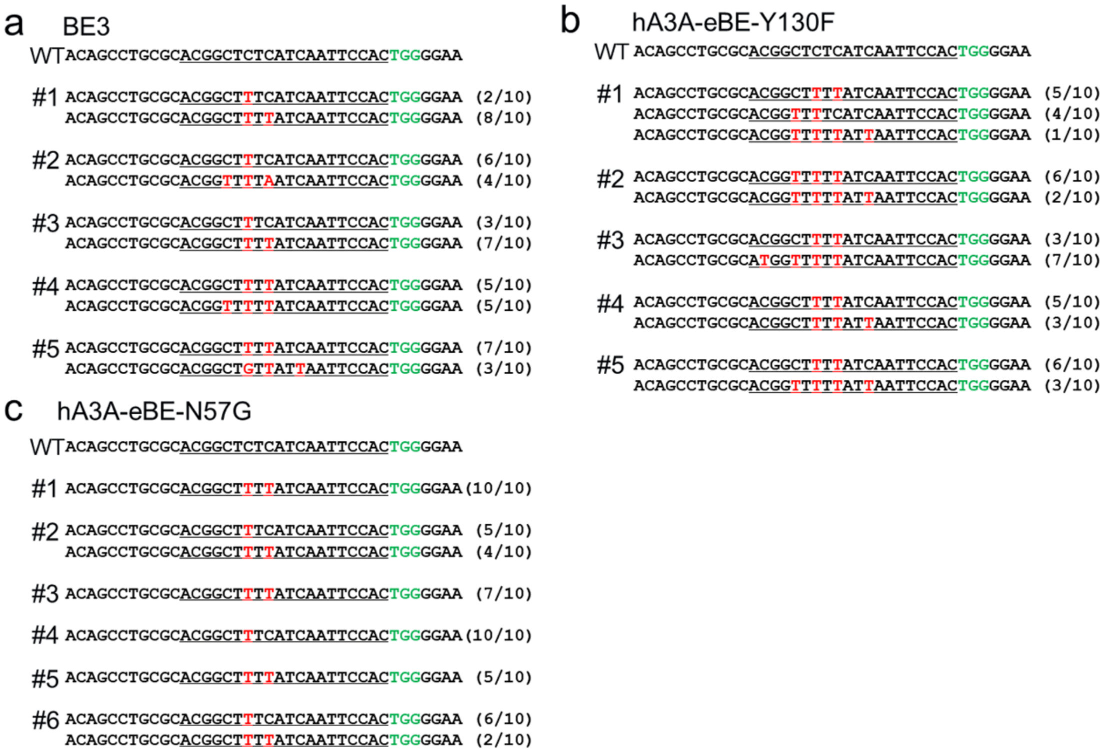

Figure S6. Comparison of the editing efficiencies of BE3 and A3A-Cas9 fusions at *Lmna-2* in rabbit blastocysts. The number of clones for each sequence pattern is indicated. The target sequence (underlined), PAM region (green) and substituted nucleotides (red) are shown. WT: wild-type. #1-#5: each blastocyst used for T-A cloning.

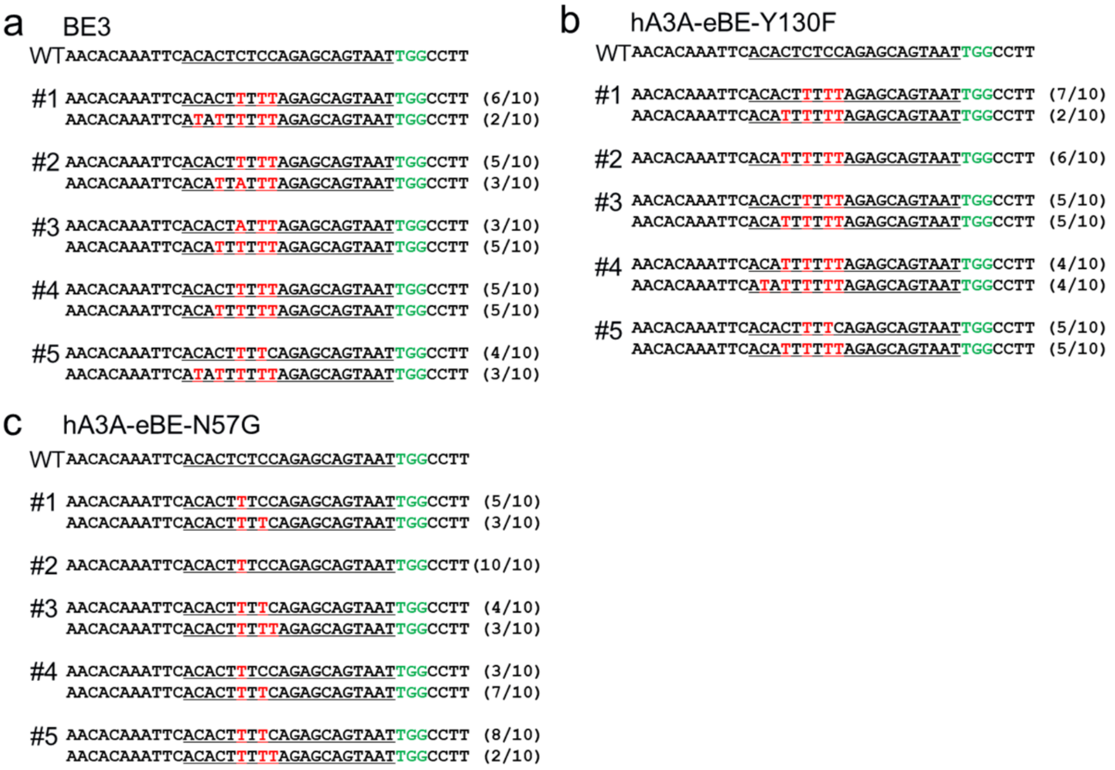

Figure S7. Comparison of the editing efficiencies of BE3 and A3A-Cas9 fusions at *Mstn* in rabbit blastocysts. The number of clones for each sequence pattern is indicated. The target sequence (underlined), PAM region (green) and substituted nucleotides (red) are shown. WT: wild-type. #1-#5: each blastocyst used for T-A cloning.

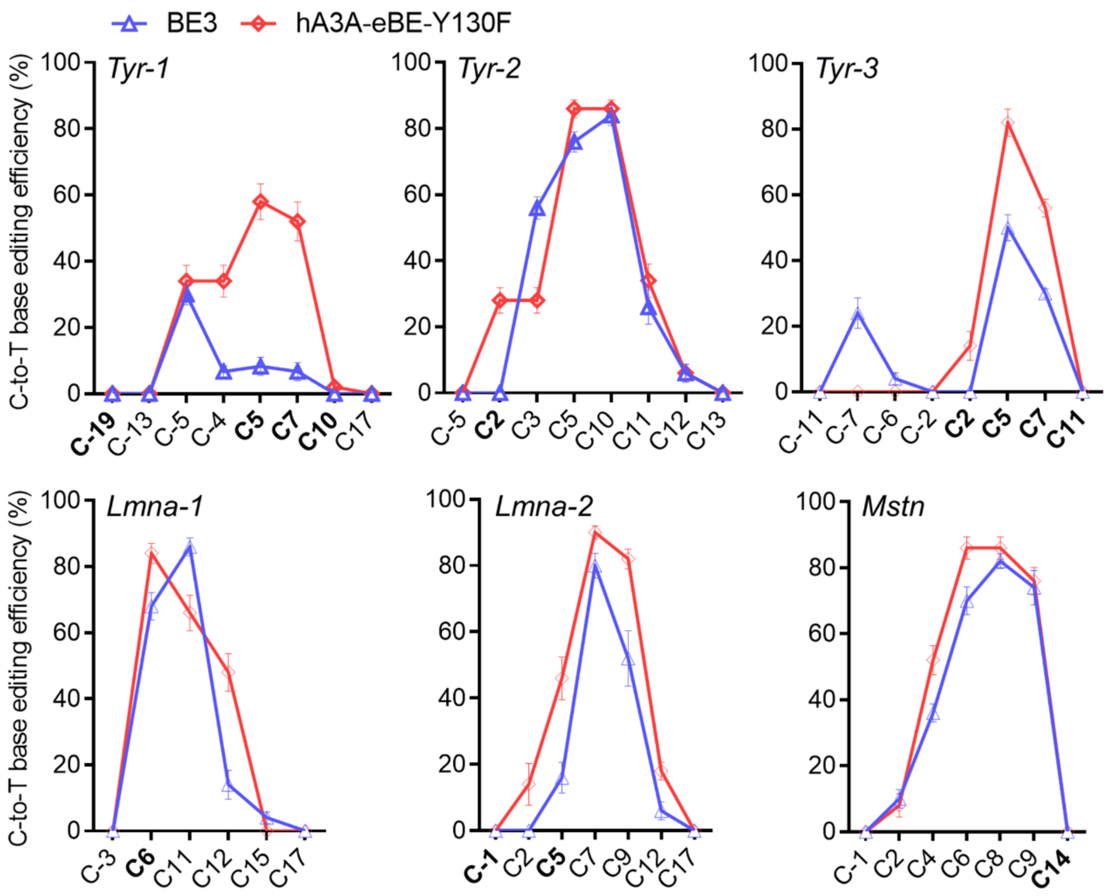

Figure S8. Frequencies of single C-to-T conversions using BE3 and hA3A-eBE-Y130F at six sites that included all base contexts in rabbit blastocysts. GC contexts are indicated in bold on the x axis.

	Sequence	Frequency (%)	
WT	CCCCTTGGGCCTCAATTCCCCTTCACAGGGGTGGATGAA		
AT1	CCCCTTGGGCCTTAATTCCCCTTCACAGGGGTGGATGAA	68	Q68Stop
	CCCCTTGGGCCTTAATTTCCCTTCACAGGGGTGGATGAA	23	Q68Stop,F69F
	CCCCTTGGGCCTTAATTGCCCTTCACAGGGGTGGATGAA	3	Q68Stop,F69L
	CCCCTTGGGCCTTAATTACCCTTCACAGGGGTGGATGAA	3	Q68Stop,F69L
	CCCCTTGGGCTTTAATTCCCCTTCACAGGGGTGGATGAA	2	P67L,Q68Stop
AT2	CCCCTTGGGCCTTAATTCCCCTTCACAGGGGTGGATGAA	60	Q68Stop
	CCCCTTGGGTCTTAATTCCCCTTCACAGGGGTGGATGAA	20	P67S,Q68Stop
	CCCCTTGGGCCTTAATTTCCCTTCACAGGGGTGGATGAA	16	Q68Stop,F69F
	CCCCTTGGGTCTTAATTTCCCTTCACAGGGGTGGATGAA	1	P67S,Q68Stop,F69F
AT3	CCCCTTGGGCCTTAATTCCCCTTCACAGGGGTGGATGAA	54	Q68Stop
	CCCCTTGGGCCTTAATTTCCCTTCACAGGGGTGGATGAA	20	Q68Stop,F69F
	CCCCTTGGGTCTTAATTCCCCTTCACAGGGGTGGATGAA	17	P67S,Q68Stop
	CCCCTTGGGGCTTAATTCCCCTTCACAGGGGTGGATGAA	4	P67A,Q68Stop
	CCCCTTGGGCTTTAATTCCCCTTCACAGGGGTGGATGAA	2	P67L,Q68Stop
	CCCCTTGGGTCTTAATTTCCCTTCACAGGGGTGGATGAA	1	P67S,Q68Stop,F69F
AT4	CCCCTTGGGCCTTAATTCCCCTTCACAGGGGTGGATGAA	92	Q68Stop
	CCCCTTGGGCCTTAATTACCCTTCACAGGGGTGGATGAA	4	Q68Stop,F69L
	CCCCTTGGGCCTTAATTTCCCTTCACAGGGGTGGATGAA	1	Q68Stop,F69F
AT5	CCCCTTGGGCCTTAATTCCCCTTCACAGGGGTGGATGAA	75	Q68Stop
	CCCCTTGGGCCTTAATTTCCCTTCACAGGGGTGGATGAA	21	Q68Stop,F69F
	CCCCTTGGGCCTTAATTACCCTTCACAGGGGTGGATGAA	1	Q68Stop,F69L
	CCCCTTGGGCCT-----CCCCTTCACAGGGGTGGATGAA	1	-5bp

Figure S9. Alignments of mutant sequences from deep sequencing at *Tyr* Q68Stop using hA3A-eBE-N57G. The targeted sequence is underlined. The PAM site and base conversions are shown in green and red, respectively. The column on the right indicates frequencies of mutant alleles. WT, wild-type. AT1-AT5, each individual.

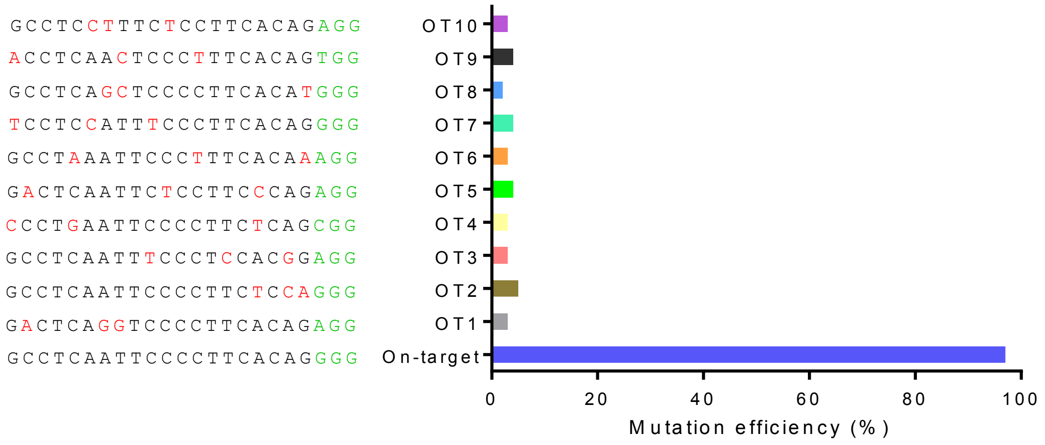

Figure S10. Off-target detection by deep sequencing in the representative F0 mutant rabbit (AT4). The PAM and mismatched nucleotides are shown in green and red, respectively.

Supplementary Tables

Table S1. Summary of the *Tyr* p.Q68Stop rabbits generated using the hA3A-eBE-N57G system.

Target site	Embryos transferred	No. of offspring	Mutant ratio (%)			
			No. of mutants	Frequencies of target p.Q68Stop	Frequencies of bystander mutations	Pups with colour change
Tyr p.Q68Stop	40	5	5(100)	(97.60 ± 0.40)	(27.80 ± 6.75)	5(100)

Table S2. Primers used for genotyping and site-directed mutation in this study. The N57G mutation sites are shown in red.

Target site	Primers	Sequence (5'-3')	Product size (bp)
Tyr-1	*Tyr-1*-F<br>*Tyr-1*-R	GTGGTGGATGCAAGACTAGAA<br>AGCTGAAATTGGCAGCTTTG	408
Tyr-2	*Tyr-2*-F<br>*Tyr-2*-R	ATCCGCTCAAGCAGGTATTG<br>GACATAGTCTGGGCTCGTAGTA	487
Tyr-3	*Tyr-3*-F<br>*Tyr-3*-R	TGATGATGGCAGTAGTGGTAATG<br>CCAGTCTATTGGGCTTCTCTTG	418
Lmna-1	*Lmna -1*-F<br>*Lmna -1*-R	AGCACCCAAGCCACAAAT<br>CACAAAGGCATGACTCACCT	490
Lmna-2	*Lmna-2*-F<br>*Lmna-2*-R	ACAGTAGCACGGAGTGAGT<br>TAGGAGACCGGCAAAGTGA	237
Mstn	*Mstn* -F<br>*Mstn* -R	TAGAGGTCAAGGTAACGGACA<br>GAGACATCTTTGTGGGAGTACAG	282
N57G introduced into hA3A	N57G-F<br>N57G-R	GGGGCTTTCTACACGGCCAGGCTAAGAATC<br>CCGTGTAGAAAGCCCCTGTGCTGGTCCATC	

Table S3. The two oligonucleotide strands used to construct the pUC57-sgRNA vectors.

**Target site**	**Oligonucleotide 1**	**Oligonucleotide 2**
Tyr-1	TAGGATTGCGCAGCAAGGGTCCCT	AAACAGGGACCCTTGCTGCGCAAT
Tyr-2	TAGGCCTCAATTCCCCTTCACAG	AAACCTGTGAAGGGGAATTGAGG
Tyr-3	TAGGCAGCGCTGGCCACACTCAT	AAACATGAGTGTGGCCAGCGCTG
Lmna-1	TAGGTGGGCGGATCCATCTCCTC	AAACGAGGAGATGGATCCGCCCA
Lmna-2	TAGGACGGCTCTCATCAATTCCAC	AAACGTGGAATTGATGAGAGCCGT
Mstn	TAGGACACTCTCCAGAGCAGTAAT	AAACATTACTGCTCTGGAGAGTGT

Table S4. The primers used for identifying potential off-target sites in this study. The mismatched nucleotides are shown in red.

Potential Off Target Site	Number of mismatches	Position	PCR Primer
GACTCAGGTCCCCTTCACAG AGG	3	chr13:-134185543	OT1-F: TTCTCTCCTCTCCGCATTCT<br>OT1-R: TGATGTCAGAGGCCGTCTAT
GCCTCAATTCCCCTTCTCCA GGG	3	chr14:-32031659	OT2-F: CCACAAGCCCTGTAGAATCA<br>OT2-R: GGAACGCTTATGCCCATTTC
GCCTCAATTTCCCTCCACGG AGG	3	chr16:-44146399	OT3-F: GCAATAGCTTTGTGGCCTTG<br>OT3-R: ACCCATGTGAGATGCTGATG
CCCTGAATTCCCCTTCTCAG CGG	3	chr2:+112817702	OT4-F: CAGAGGAGACAAAGCAACAATTC<br>OT4-R: CCCGTCACCATCCCATTAC
GACTCAATTCTCCTTCCCAG AGG	3	chr3:+55437364	OT5-F: CAGGCTCAAAGGTGGTATGA<br>OT5-R: GAGGACTCTGTGCTTGTTAGAG
GCCTAAATTCCCTTTCACAA AGG	3	chr5:-17790033	OT6-F: TGCCTGTGGGTAATGGTTAG<br>OT6-R: CCCTAGTCTGAGTGCCAATTTA
TCCTCCATTTCCCTTCACAG GGG	3	chr7:-99342691	OT7-F: CACTCGTGGAGTTTCCTGAAT<br>OT7-R: AGAAGAAGAAGAGGAGGAGGAG
GCCTCAGCTCCCCTTCACAT GGG	3	chr9:-21114580	OT8-F: TGCCATCACCTAAAGGCTAAG<br>OT8-R: TGTGGGTCTGTAAGCTCTTTG
ACCTCAACTCCCTTTCACAG TGG	3	chr9:-29521405	OT9-F: AGCTGGGTGATAGGGATACA<br>OT9-R: CCAACCGAGCCTGGATATTT
GCCTCCTTTCTCCTTCACAG AGG	3	chrX:-46475662	OT10-F: TCTGAAAGGGAGTCAAGGAAAC<br>OT10-R: TCTGCCAACAGCCTGAAAT
